# Bats: Secrets of Extended Healthspan

**DOI:** 10.1093/geroni/igab046.1590

**Published:** 2021-12-17

**Authors:** Emma Teeling

**Affiliations:** University College Dublin, Ireland, Ireland

## Abstract

Of all mammals, bat possess the most unique and peculiar adaptations that render them as excellent models to investigate the mechanisms of extended longevity and potentially halted senescence. Indeed, they are the longest-lived mammals relative to their body size, with the oldest bat caught being >41 years old, living approx. 8 times longer than expected. Bats defy the ‘rate-of-living’ theories that propose a positive correlation between body size and longevity as they use twice the energy as other species of considerable size, but live far longer. The mechanisms that bats use to avoid the negative physiological effects of their heightened metabolism and deal with an increased production of deleterious Reactive Oxygen Species (ROS) is not known, however it is suggested that they either prevent or repair ROS damage. Bats also appear to have resistance to many viral diseases such as rabies, SARS and Ebola and are the suspected reservoir species for a huge diversity of newly discovered viruses, including Sars-CoV-2 This suggests that their innate immunity is different to other mammals, perhaps playing a role in their unexpected longevity. Here the potential genomic basis for their rare immunity and exceptional longevity is explored across multiple bat genomes and divergent ageing and immune related markers (e.g. microbiome, telomeres, mitochondria, cellular dynamics, cytokine response) studied in wild bat populations. These findings provide a deeper understanding of the causal mechanisms of ageing and tolerant immunity, potentially uncovering the key molecular pathways that could be utilised to benefit society.

